# Lymphocyte trajectories are associated with prognosis in critically ill patients: A convenient way to monitor immune status

**DOI:** 10.3389/fmed.2022.953103

**Published:** 2022-08-04

**Authors:** Fei Pei, Wenliang Song, Luhao Wang, Liqun Liang, Bin Gu, Minying Chen, Yao Nie, Yishan Liu, Yu Zhou, Xiangdong Guan, Jianfeng Wu

**Affiliations:** ^1^Department of Critical Care Medicine, The First Affiliated Hospital, Sun Yat-sen University, Guangzhou, China; ^2^Guangdong Clinical Research Center for Critical Care Medicine, Guangzhou, China; ^3^Clinical Trials Unit, The First Affiliated Hospital, Sun Yat-sen University, Guangzhou, China

**Keywords:** lymphopenia, immunosuppression, lymphocyte (LYM), critically ill patient, persistent inflammation immunosuppression and catabolism syndrome

## Abstract

**Background:**

Immunosuppression is a risk factor for poor prognosis of critically ill patients, but current monitoring of the immune status in clinical practice is still inadequate. Absolute lymphocyte count (ALC) is not only a convenient biomarker for immune status monitoring but is also suitable for clinical application. In this study, we aimed to explore different trajectories of ALC, and evaluate their relationship with prognosis in critically ill patients.

**Methods:**

We retrospectively enrolled 10,619 critically ill patients admitted to a general intensive care unit (ICU) with 56 beds from February 2016 to May 2020. Dynamic ALC was defined as continuous ALC from before ICU admission to 5 days after ICU admission. Initial ALC was defined as the minimum ALC within 48 h after ICU admission. Group-based trajectory modeling (GBTM) was used to group critically ill patients according to dynamic ALC. Multivariate cox regression model was used to determine the independent association of trajectory endotypes with death and persistent inflammation, immunosuppression, catabolism syndrome (PICS).

**Results:**

A total of 2022 critically ill patients were unsupervisedly divided into four endotypes based on dynamic ALC, including persistent lymphopenia endotype (*n* = 1,211; 58.5%), slowly rising endotype (*n* = 443; 22.6%), rapidly decreasing endotype (*n* = 281; 14.5%) and normal fluctuation endotype (*n* = 87; 4.4%). Among the four trajectory endotypes, the persistent lymphopenia endotype had the highest incidence of PICS (24.9%), hospital mortality (14.5%) and 28-day mortality (10.8%). In multivariate cox regression model, persistent lymphopenia was associated with increased risk of 28-day mortality (HR: 1.54; 95% CI: 1.06–2.23), hospital mortality (HR: 1.66; 95% CI: 1.20–2.29) and PICS (HR: 1.79; 95% CI: 1.09–2.94), respectively. Sensitivity analysis further confirmed that the ALC trajectory model of non-infected patients and non-elderly patients can accurately distinguished 91 and 90% of critically ill patients into the same endotypes as the original model, respectively.

**Conclusion:**

The ALC trajectory model is helpful for grouping critically ill patients, and early persistent lymphopenia is associated with poor prognosis. Notably, persistent lymphopenia may be a robust signal of immunosuppression in critically ill patients.

## Introduction

Immunosuppression is a risk factor for death in critically ill patients (1). Unfortunately, there are still no guidelines for clinical immune function monitoring. Monocyte human leukocyte antigen-DR (mHLA-DR), absolute lymphocyte count (ALC), and lymphocyte subsets, such as CD4/CD8 and regulatory T cell (Treg), are suitable biomarkers for evaluating the immune function in critically ill patients, such as those with trauma, those who recently had an operation and those with sepsis (2–4). Given that lymphocyte subsets and mHLA-DR need to be measured by flow cytometry, they are difficult to detect in real time in many hospitals (5). Unlike other biomarkers, ALC is obtained from routine blood analysis and is thus an easily accessible biomarker of the immune status of critically ill patients.

According to a large retrospective cohort study, lymphopenia increases the risk of infection and infection-related death in the general population (6). In patients with sepsis, lymphopenia is associated with increased mortality, and dynamic lymphocyte decline is also associated with poor prognosis (7–10). Nevertheless, due to the heterogeneity of critically ill patients, there are also differences in ALC; for example, ALC at admission to ICU is lower in elderly patients than in non-elderly patients (11). One way to understand this heterogeneity is to divide critically ill patients into different endotypes by unsupervised classification, whereafter it is helpful to identify the association between endotypes and adverse clinical outcomes. Group-based trajectory modeling (GBTM) is a new methodology for studying the trajectory of biomarkers over time (12, 13). It has been proven to be a robust methodology for capturing longitudinal adherence and predicting biomarkers and clinical events in various areas (14, 15). In COVID-19 patients, GBTM was used to group convalescent patients by CT and lymphocytes, and it showed that patients with different severity of disease had different trajectories (16). Therefore, GBTM is a suitable method for the clinical classification of critically ill patients.

The aim of this retrospective study was to group critically ill patients according to ALC over time, and to assess the relationship between different trajectory endotypes and outcomes in critically ill patients.

## Materials and methods

### Study design and settings

This study was a single-center retrospective clinical study based on an electronic database from the Hospital Information System from February 2016 to May 2020 in a tertiary care general ICU with about 2,000 admissions per year. The study protocol was approved by the Independent Ethics Committee for clinical research and animal trials of the First Affiliated Hospital of Sun Yat-sen University [IEC no. (2020) 266]. Adult patients (aged 18 years or older) with continuous routine blood analysis more than 4 days after admitting to the ICU were enrolled, and patients with abnormally high lymphocyte counts were excluded. Demographic data, clinical data and follow-up data were available in the electronic database.

### Study definitions

Baseline ALC was defined as the mean ALC of ALCs between 7 days before ICU admission and 24 h after ICU admission, and Day n was defined as the mean ALC from the n day to the n+1 day (Supplementary Figure 1). The diagnosis of persistent inflammation, immunosuppression and catabolism syndrome (PICS) was based on the following criteria: the length of ICU stay more than 14 days; C-reactive protein over than 30 mg/L, ALC less than 0.8 × 10^9^/L, and body mass index less than 18 or pre-albumin level less than 100 mg/L or albumin level less than 30 mg/L on the 14th day after ICU admission (17, 18). Of note, if a patient received an infusion of human serum albumin, albumin concentration was discarded. An infected patient was defined as a critically ill patient in whom pneumonia, abdominal infection, skin and soft tissue infection, urinary tract infection, intracranial infection, catheter-related infection, or blood infection was diagnosed. Sepsis was diagnosed accordance with Sepsis-3 criteria (19). Acute kidney injury (AKI) was defined as serum creatinine level within the previous 7 days that was 1.5 times higher than at baseline, or urine volume less than 0.5 mL/kg/h for 6 h (20). Acute physiology, age and chronic health evaluation II (APACHE-II) score was assessed on ICU admission day.

According to a previous study (21), normal ALC was defined as at least 1.1 × 10^9^/L, and lymphopenia was divided into two categories: mild lymphopenia (0.7–1.1 × 10^9^/L) and severe lymphopenia (≤ 0.7 × 10^9^/L). Initial severe lymphopenia was defined as severe lymphopenia within 48 h after ICU admission. Because ALC did not conform to the normal distribution, the original data were converted to the approximate normal distribution data using Box-Cox method.

### Statistical methods

Demographics, comorbidities and physiological characteristics (i.e., laboratory results) processes of care and outcomes were compared among the identified trajectory groups. For these comparisons, continuous variables were tested using one-way ANOVA for normal distributions while Kruskal-Wallis *H*-test for non-normal distributions; categorical variables were tested using χ^2^-test or Fisher exact test, as appropriate. Generalized estimating equation was applied to test difference of ALC between survivors and non-survivors. Kaplan–Meier curve was depicted and log-rank test was applied to test the hypothesis. Cox regression was used to determine the hazard ratio (HR) and 95% confidence interval (95% CI) for the association of ALC trajectory endotypes, adjusted for the APACHE-II score, age (≥ 60 years, < 60 years), initial severe lymphopenia, sex, cancer, hypertension, diabetes, AKI, and infection. Owing that death prevents the occurrence of PICS, competing risks model, was applied to reduce the bias while the occurrence of PICS and death were defined as the event of interest and competing event, respectively (22).

GBTM is a specialized application of finite mixture modeling and is used to identify groups of individuals following similar trajectories for a particular variable of interest (12, 23). This model was used to identify latent endotypes based on ALC trajectories from before ICU admission to 5 days after ICU admission. The Bayesian information criterion (BIC) was used for model selection, which penalizes more complex models to ensure that a well-fitting yet parsimonious model is chosen (13, 24). Two-, three-, four-, and five-group models, with linear, quadratic, cubical and quartic functions were explored to select the most suitable fit among the population. We also performed sensitivity analysis in elderly/non-elderly patients and infected/non-infected patients to explore whether this trajectory model is suitable for patients at a high risk of immunosuppression. Two-tailed *P*-values less than 0.05 were considered statistically significant for all hypothesis tests. All analyses were performed using Stata version 15.1.

## Results

### Characteristics of critically ill patients

**FIGURE 1:**
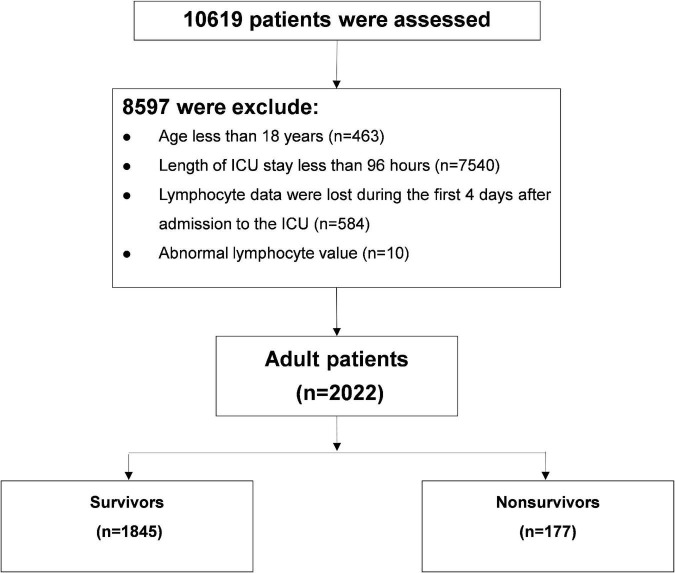
Flow chart of critically ill patients.

**FIGURE 2:**
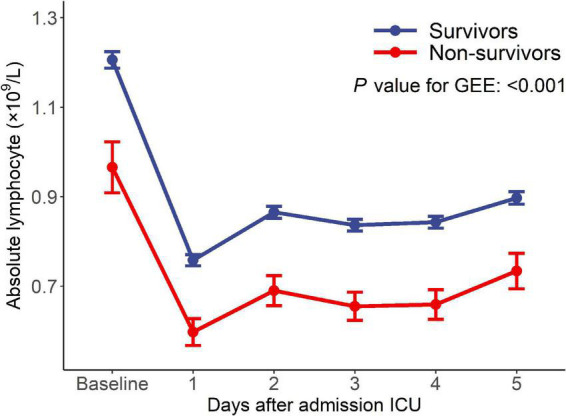
Dynamic changes of ALC between the survivors and the non-survivors.

Of 10,619 critically ill patients, 2022 patients with continuous ALC monitoring were included in this study (Figure 1). There were 1,845 critically ill patients in the survival group and 177 patients in the non-survival group. The ALC in the survival group was higher than that in the non-survival group (*P*-value of generalized estimating equation, GEE: *P* < 0.001), and the dynamic trend was similar in the two groups after admission to ICU (Figure 2).

### Trajectories of absolute lymphocyte count in critically ill patients

**FIGURE 3:**
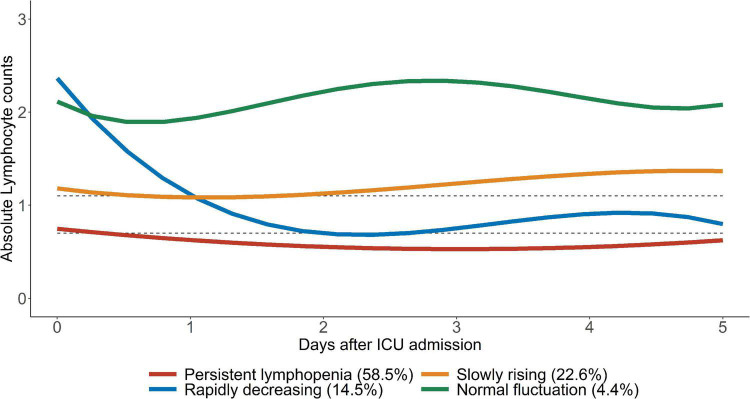
Trajectories of ALC in critically ill patients. Four ALC trajectory endotypes were grouped in critically ill patients: persistent lymphopenia endotype, slowly rising endotype, rapidly decreasing endotype and normal fluctuation endotype. The upper gray dotted line represents 1.1 × 10^9^/L and the lower gray dotted line represents 0.7 × 10^9^/L.

By comparing the posterior probability and fitting degree of group number and shape in GBTM, a four-trajectory model with the lowest BIC was constructed (Figure 3). Group 1 (*n* = 1,211; 58.5%) was characterized by severe lymphopenia that lasted for 5 days after admission to ICU (hereafter defined as “persistent lymphopenia”). Unlike group 1, group 2 (*n* = 443; 22.6%) presented normal ALC at admission to ICU, but the ALC declined rapidly to mild lymphopenia on day 2 (hereafter defined as “rapidly decreasing”). Group 3 (*n* = 281; 14.5%) presented initial marginal mild lymphopenia at admission to ICU, and the ALC then gradually increased to normal from day 3 (hereafter defined as “slowly rising”). Finally, ALC in group 4 (*n* = 87; 4.4%) was fluctuated around the normal value before and after admission to ICU (hereafter defined as “normal fluctuation”).

Demographics, comorbidities and physiological characteristics of the four ALC trajectory endotypes were summarized in Table 1. The persistent lymphopenia endotype had the highest age [median 61 years; interquartile range (IQR) 49–70], APACHE-II scores (median 19; IQR 15–24) and CRP level (median 58; IQR 14–132). In addition, patients with persistent lymphopenia had the highest incidence of sepsis (376/1,211; 31.0%) and AKI (454/1,211; 37.5%). Similar to the persistent lymphopenia endotype, the median age of the rapidly decreasing endotype was 59 years (IQR: 48–66); however, the incidence rates of sepsis (42/281; 14.9%) and AKI (79/281; 28.1%) were lower in rapidly decreasing patients. In the slowly rising endotype, the APACHE score (median 18; IQR 13–22) and CRP level (median 52; IQR 14–130) were similar to those in the persistent lymphopenia endotype, while patients in the slowly rising endotype were younger (median 54 years; IQR 43–66).

**TABLE 1:** Baseline characteristics in four ALC trajectory endotypes.

Characteristic	Persistent lymphopenia (*N* = 1,211)	Slowly rising (*N* = 443)	Rapidly decreasing (*N* = 281)	Normal fluctuation (*N* = 87)	*P*-value
Age, year—Median (IQR)	61 (49–70)	54 (43–66)	59 (48–66)	44 (33–59)	<0.001
Sex, male—no. (%)	819 (67.6)	283 (63.9)	179 (63.7)	58 (66.7)	0.397
APACHE II score—Median (IQR)	19 (15–24)	18 (13–22)	17 (13–21)	14.5 (11–21)	<0.001
**Preexisting condition—no. (%)**					
Hypertension	345 (28.5)	153 (34.5)	91 (32.4)	20 (23.0)	0.038
Coronary heart disease	99 (8.2)	45 (10.2)	36 (12.8)	8 (9.2)	0.097
Diabetes	172 (14.2)	78 (17.6)	41 (14.6)	15 (17.2)	0.349
Chronic renal failure	90 (7.4)	16 (3.6)	12 (4.3)	5 (5.7)	0.017
Cancer	430 (35.5)	83 (18.7)	135 (48.0)	14 (16.1)	<0.001
**Complications—no. (%)**					
Sepsis	376 (31.0)	101 (22.8)	42 (14.9)	22 (25.3)	<0.001
Infection *	697 (57.6)	242 (54.6)	99 (35.2)	51 (58.6)	<0.001
Acute pancreatitis	55 (4.5)	31 (7.0)	1 (0.4)	6 (6.9)	<0.001
ARDS	41 (3.4)	16 (3.6)	2 (0.7)	3 (3.4)	0.105
AKI	454 (37.5)	112 (25.3)	79 (28.1)	18 (20.7)	0.001
Mechanical ventilation—no. (%)	703 (58.1)	249 (56.2)	166 (59.1)	40 (46.0)	0.144
RRT—no. (%)	273 (22.5)	76 (17.2)	30 (10.7)	14 (16.1)	<0.001
Vasopressors—no. (%)	1,011 (83.5)	343 (77.4)	220 (78.3)	53 (60.9)	<0.001
Initial lymphocyte (10^9^/L),—Median (IQR)	0.72 (0.43–1.1)	1.20 (0.90–1.53)	2.33 (2.06–2.72)	1.99 (1.62–2.63)	<0.001
CRP (mg/L),—Median (IQR)	58 (14–132)	52 (14–130)	17 (4–66)	52 (17–109)	<0.001
ICU stay days,—Median (IQR)	9 (6–14)	10 (6–18)	8 (6–12)	9 (6–17)	<0.001

Values are described by number (%) or median (interquartile range).

*Infection includes pneumonia, abdominal infection, skin and soft tissue infection, urinary tract infection, intracranial infection, catheter-related infection, and blood infection. AKI, acute kidney injury; APACHE II, acute physiology and chronic health evaluation II; ARDS, acute respiratory distress syndrome; CRP, C-reactive protein; RRT, renal replacement therapy.

Previous studies have demonstrated the persistent lymphopenia usually present in infected and elderly patients (8, 11). To evaluate the applicability of this ALC trajectory model in all critically ill patients, sensitivity analysis was performed to confirm whether this model was appropriate for non-infected patients and non-elderly patients. In our study, the ALC trajectory models of non-infected patients and non-elderly patients were similar to the original model, and these models classified 91 and 90% of the patients into the same groups as the original model, respectively (Supplementary Figure 2).

### Outcome comparison between the absolute lymphocyte count trajectory endotypes

**FIGURE 4:**
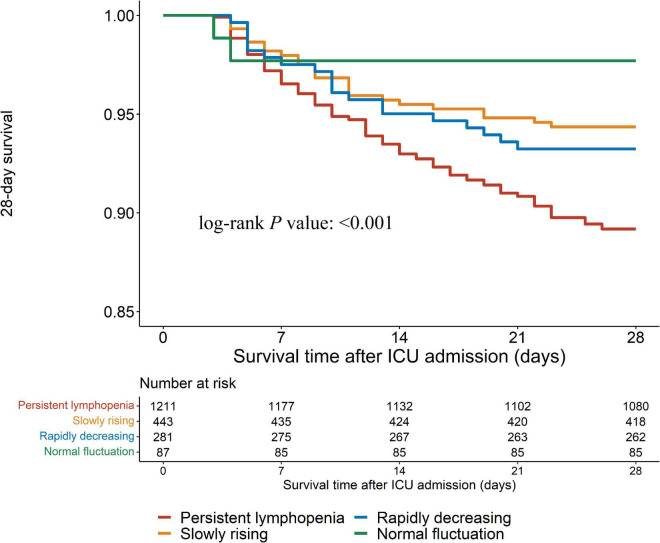
Kaplan-Meier curves for 28-day mortality.

**FIGURE 5:**
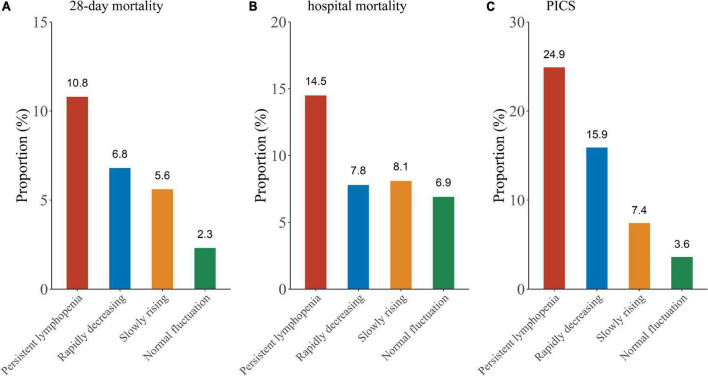
Outcome comparison between the ALC trajectory endotypes. **(A)** Twenty-eight-day mortality; **(B)** hospital mortality; **(C)** PICS.

In this study, outcomes were assessed by 28-day mortality, hospital mortality and the incidence of PICS. Log-rank test showed that the persistent lymphopenia endotype had the highest incidence of the 28-day mortality among the four endotypes (Figures 4, 5A; *P* < 0.001). Similarly, the persistent lymphopenia endotype also had the highest hospital mortality (Figure 5B). As for the incidence of PICS between the ALC trajectory endotypes, a quarter of patients had PICS in the persistent lymphopenia endotype (92/370; 24.9%), which was about seven times than that in the normal fluctuation endotype (1/28; 3.6%), about three times higher than that in the slowly rising endotype (13/175; 7.4%) and about 1.5 times higher than that in the rapidly decreasing endotype (10/63; 15.9%) (Figure 5C). Furthermore, we found that patients in the persistent lymphopenia endotype also had higher CRP levels and lower pre-albumin levels after admission to ICU than other endotype patients (Supplementary Figure 3).

We found that more than a quarter (27.2%) of infected patients in the persistent lymphopenia endotype developed PICS, which was higher compared with non-infected (17.8%) patients (Supplementary Figures 4A,B). Similarly, the incidence of PICS in elderly patients of the persistent lymphopenia endotype was higher than that in non-elderly patients (30.9 vs. 18.6%) (Supplementary Figures 4C,D). In terms of mortality, patients in persistent lymphopenia endotype also had the highest 28-day mortality in non-infected subgroup and elderly subgroup (Supplementary Figure 5).

### Persistent lymphopenia is associated with persistent inflammation, immunosuppression, catabolism syndrome, and death

Previous studies have found that severe lymphopenia and dynamic changes in ALC are associated with mortality in critically ill patients (8, 25, 26). The ALC trajectory endotypes was taken as a binary variable. Three other endotypes were combined as control to evaluate the association between persistent lymphopenia and prognosis. After adjusting for the APACHE-II score, age, sex, initial severe lymphopenia, cancer, hypertension, diabetes, AKI and infection, persistent lymphopenia was associated with increased risk of hospital mortality (HR: 1.66; 95% CI: 1.20–2.29), 28-day mortality (OR: 1.54; 95% CI: 1.06–2.23) and PICS (OR: 1.79; 95% CI: 1.09–2.94) in critically ill patients, respectively (Table 2). In a competing risks model, persistent lymphopenia was related with higher risk of the occurrence of PICS (sHR: 1.90; 95% CI: 1.14–3.15).

**TABLE 2:** Hazard ratios estimates for PICS and death from multivariate regression modeling.

Outcomes	HR/sHR (95% CI)	*P*-value
**28-Day mortality**		
Persistent lymphopenia**^#^**	1.54 (1.06–2.23)	0.023
**Hospital mortality**		
Persistent lymphopenia	1.66 (1.20–2.29)	0.002
**PICS**		
Persistent lymphopenia	1.79 (1.09–2.94)	0.022
Competing risks analysis*		
Persistent lymphopenia	1.90 (1.14–3.15)	0.013

^#^The ALC trajectory endotypes was taken as a binary variable. In addition to the persistent lymphopenia endotypes, the other three endotypes were combined as a control.

*Competing risks analysis was performed based on the Fine-Gray model, and the occurrence of PICS and death were defined as the event of interest and competing event, respectively. Multivariate cox regression model and competing risks model were adjusted for APACHE-II score, sex, age, initial severe lymphopenia, cancer, hypertension, diabetes, AKI, and infection. Initial severe lymphopenia was defined as the ALC lower than 0.7 × 10^9^/L within 48 h after ICU admission. AKI, acute kidney injury; APACHE-II, acute physiology and chronic health evaluation II; HR, hazard ratio; sHR, sub-distribution hazard ratio.

## Discussion

In this retrospective, longitudinal, observational study, a total of 2022 critically ill patients with early dynamic ALC were unsupervisedly divided into four ALC trajectory endotypes. We found that the demographic characteristics, comorbidities, mortality, and PICS were different between the trajectory groups. It is particularly important to emphasize that three out of five critically ill patients admitted to ICU for more than 4 days present with persistent lymphopenia (≤0.7 × 10^9^/L). Persistent lymphopenia was independently associated with the poor prognosis of critically ill patients. These findings have important implications for understanding the heterogeneity of immune status by using the ALC trajectory model and may provide a convenient route for monitoring the immune status of critically ill patients.

ALC is one of the most readily available immune biomarkers in clinical practice, and it reflects the immune status of critically ill patients (6). Some studies have found that the decrease in lymphocyte count after admission to ICU is associated with high mortality in patients with trauma and hypotension (3, 26). Our study showed that critically ill patients with rapidly decreasing ALC had a poor prognosis, while patients with persistent lymphopenia had an even poorer prognosis. In the multivariate analysis, early persistent lymphopenia rather than initial severe lymphopenia was associated with poor prognosis. Recently, ALC has been recognized as a biomarker for screening patients in various immune regulation therapy studies (27, 28). The selection of suitable immunosuppressed patients is key for immunotherapy. In a randomized controlled trial of recombination interleukin (IL)-7 in septic patients with impaired immunity, a ALC lower than 0.9 × 10^9^/L was considered a vital inclusion criterion (28). In accordance with this cut-off value of ALC, the persistent lymphocyte endotype met the criteria, suggesting that over half of the critically ill patients may be in the status of immunosuppression. Thus, dynamic monitoring of ALC is necessary for critically ill patients.

Lymphopenia has been shown to be associated with poor prognosis in a variety of diseases, such as COVID-19, sepsis and cancer (21, 29). In addition, the degree of lymphopenia was also related to age and comorbidities in critically ill patients (11, 30). Due to the high heterogeneity of critically ill patients, it is appropriate to classify them by the dynamic change of ALC. GBTM is a widely recognized statistical technique for analyzing longitudinal data to identify specific endotypes of individuals with specific variables that change over time (12). Zhou et al. used GBTM to classify patients with acute pancreatitis into three trajectories groups (high, middle, and low group), and the trajectory of ALC was observed to be associated with the prognosis and development of infectious pancreatic necrosis (31). Unlike that study, patients with low lymphocyte counts in this study were further divided into the persistent lymphopenia endotype and rapid decreasing endotype, and the incidence of PICS and mortality in the persistent lymphopenia endotype were higher than those in the rapidly decreasing endotype. We hypothesized that the difference between the two ALC trajectory models was that our study included baseline ALC before admission to ICU. Lymphopenia during hospitalization increases the risk of infection and death; thus, the ALC of critically ill patients before admission to ICU should be emphasized (6, 25).

Monocyte HLA-DR is another biomarker commonly used to assess the immune status of critically ill patients (32, 33). Our previous study found that the dynamic change of mHLA-DR in the first week after the onset of sepsis was a reliable predictor of mortality in patients with sepsis (2). According to Leijte et al., the dynamic change of mHLA-DR over time, but not absolute values or static measurements, is of clinical importance in sepsis. Recently, Bodinier et al. divided patients with sepsis into four different trajectories (namely “non-improvers,” “decliners,” “improvers,” and “high expressors”) by dynamic mHLA-DR, which was similar to our trajectory model (34). Both studies found that the persistently low expression endotype had the largest number of patients (nearly three-fifths) and the worst prognosis. Therefore, the ALC trajectory model may be a simple method for monitoring the immune status of critically ill patients. Hence, persistent lymphopenia may be suitable as a warning sign of immunosuppression in ICU.

PICS is a common syndrome in critically ill patients who have been in the ICU for a long time, which is associated with the long-term prognosis of these patients (35, 36). In our study, nearly one-fifth of the patients experienced PICS, and the persistent lymphopenia endotype had the highest incidence of PICS among the four endotypes. Previous studies have shown that patients with PICS exhibit high healthcare resource utilization and low health-related quality of life (37, 38). Moreover, patients with persistent lymphopenia also show high inflammation and hypermetabolism. Therefore, persistent lymphopenia may be a suitable early predictor of PICS.

Our study still has some limitations. First, patients who had been in the ICU for less than 5 days and lacked continuous ALC within 5 days were excluded, which might have led to selection bias. However, in order to optimize our ALC trajectory model, we ensured a better fitness of this model through the integrity and time span of ALC. Second, this was a single-center retrospective study. Critically ill patients with various different diseases were included and the stability of the model was preliminarily evaluated through sensitivity analysis; however, multi-center data are needed for further verification of our model. Third, since the body weight of patients on day 14 after admission to ICU was not recorded in our database, the diagnosis of PICS in our study may be conservative.

## Conclusion

ALC trajectory is a convenient method for the monitoring the immune status of critically ill patients. Persistent lymphopenia is associated with the increased incidence of PICS and mortality in critically ill patients, which may be a suitable early warning sign of immunosuppression in critically ill patients.

## Data availability statement

The raw data supporting the conclusions of this article will be made available by the authors, without undue reservation.

## Ethics statement

The studies involving human participants were reviewed and approved by the Independent Ethics Committee for clinical research and animal trials of the First Affiliated Hospital of Sun Yat-sen University. Written informed consent for participation was not required for this study in accordance with the national legislation and the institutional requirements.

## Author contributions

FP was responsible for concept, statistical analyses, and drafted the manuscript. WS and LW assisted in the statistical analyses and the draft of the manuscript. JW and XG were responsible for concept, data collection, supervision, and edited the manuscript. All authors contributed to the design of the study and edited the manuscript, article and approved the submitted version.
