# Supplementary material for: Lymphocyte trajectories are associated with prognosis in critically ill patients: A convenient way to monitor immune status
Source: Front Med (Lausanne). 2022 Aug 4;9:953103. doi: 10.3389/fmed.2022.953103 (PMC9386077; doi:10.3389/fmed.2022.953103)
Supplement: Supplementary file 1 [file Data_Sheet_1.pdf]

**Figure S1.**

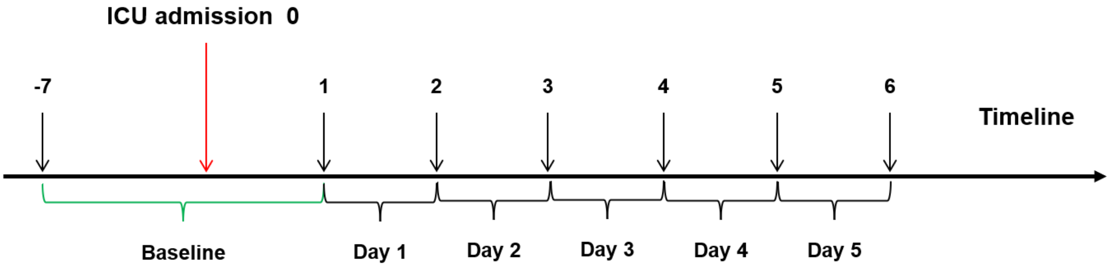


**Timeline of this study.** Baseline ALC was defined as the mean ALC of ALCs between 7 days before ICU admission and 24 hours after ICU admission, and Day n was defined as the mean ALC from the n day to the n+1 day

**Figure S2.**

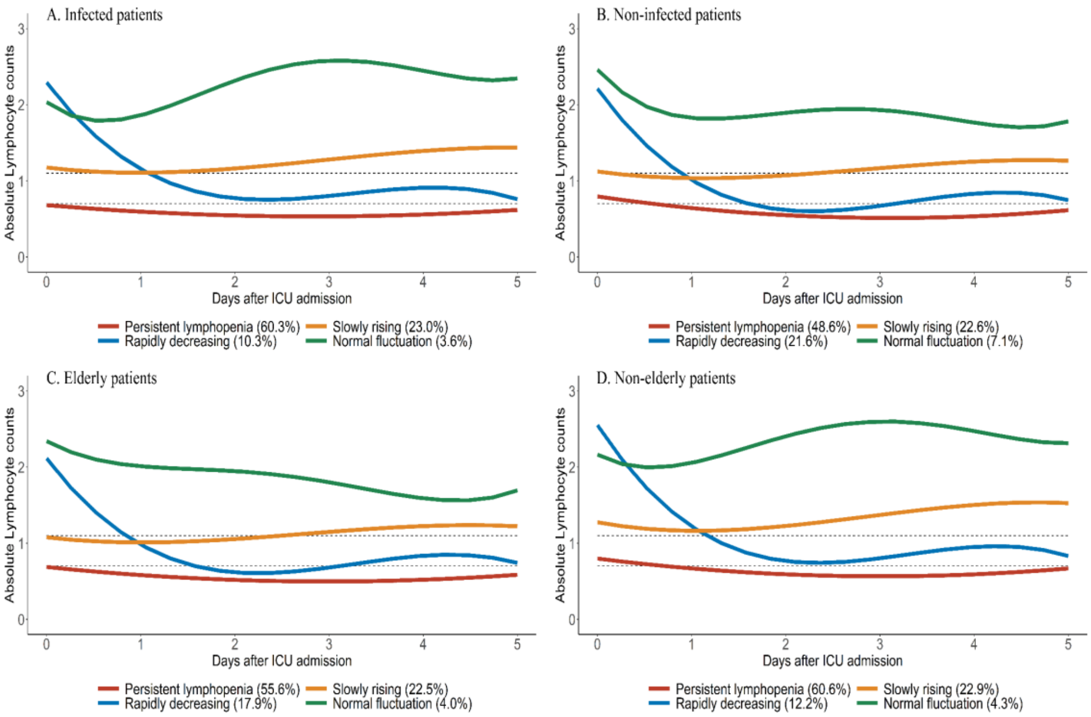


**Sensitive analysis**. A. Trajectories of ALC in infected patients; B. Trajectories of ALC in non-infected patients; C. Trajectories of ALC in elderly patients; D. Trajectories of ALC in non-elderly patients. The upper gray dotted line represents $1.1 \times 10^9$/L and the lower gray dotted line represents $0.7 \times 10^9$/L.

**Figure S3.**

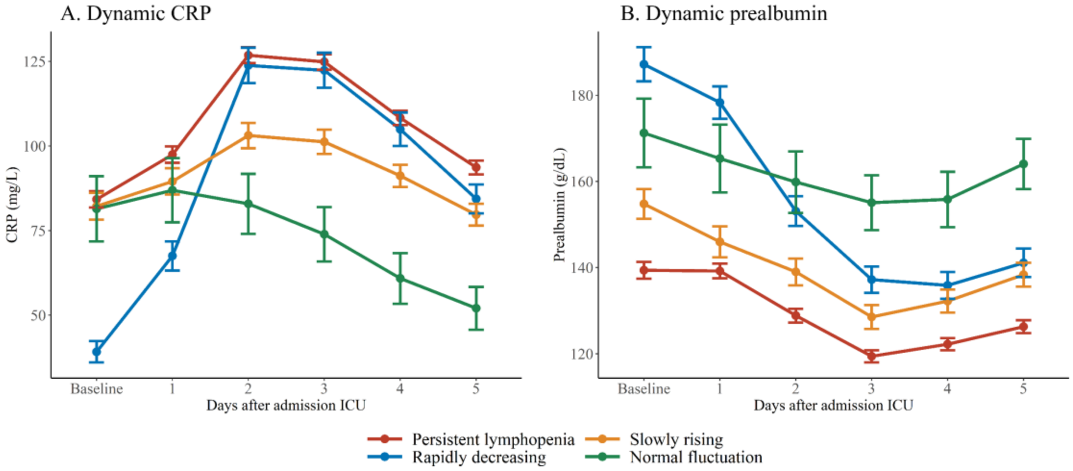


**Dynamic changes of CRP and pre-albumin among four ALC trajectory endotypes.**

**Figure S4.**

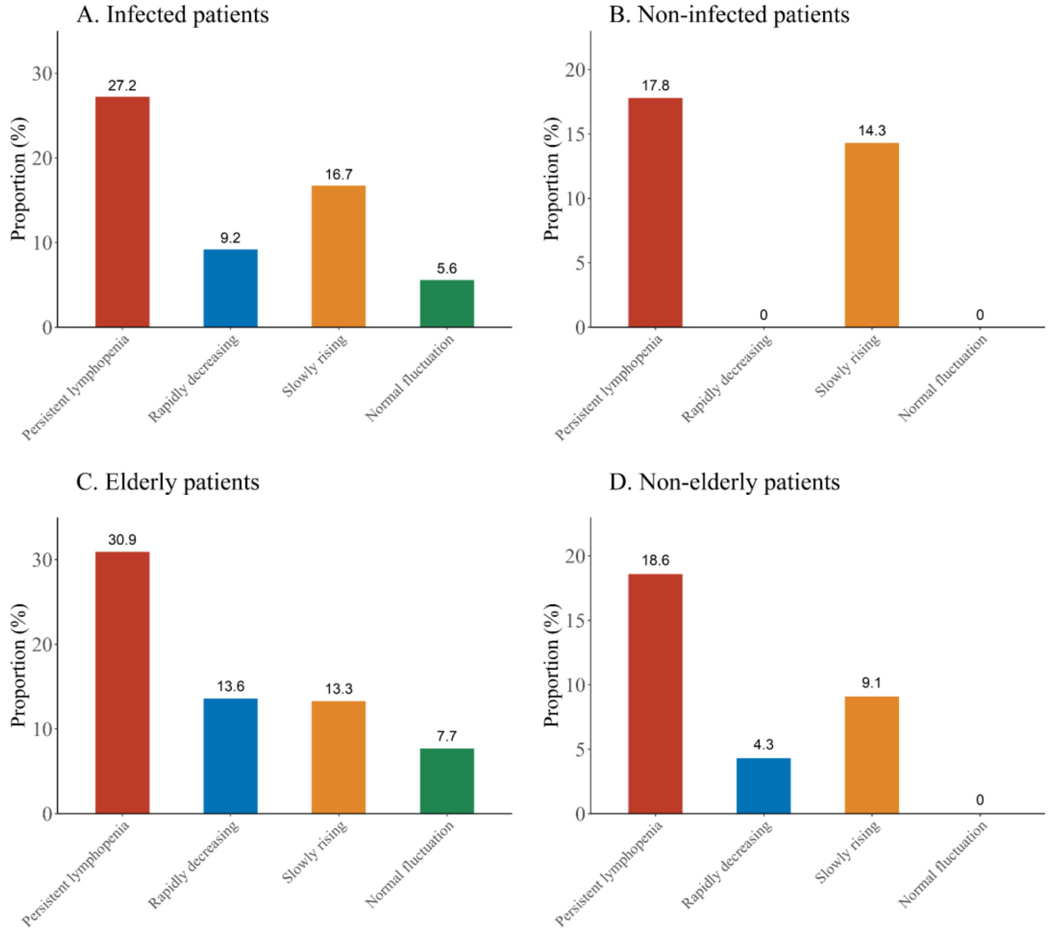


**PICS in different subgroups.** A. Infected patients; B. Non-infected patients; C. Elderly patients; D. Non-elderly patients.

**Figure S5.**

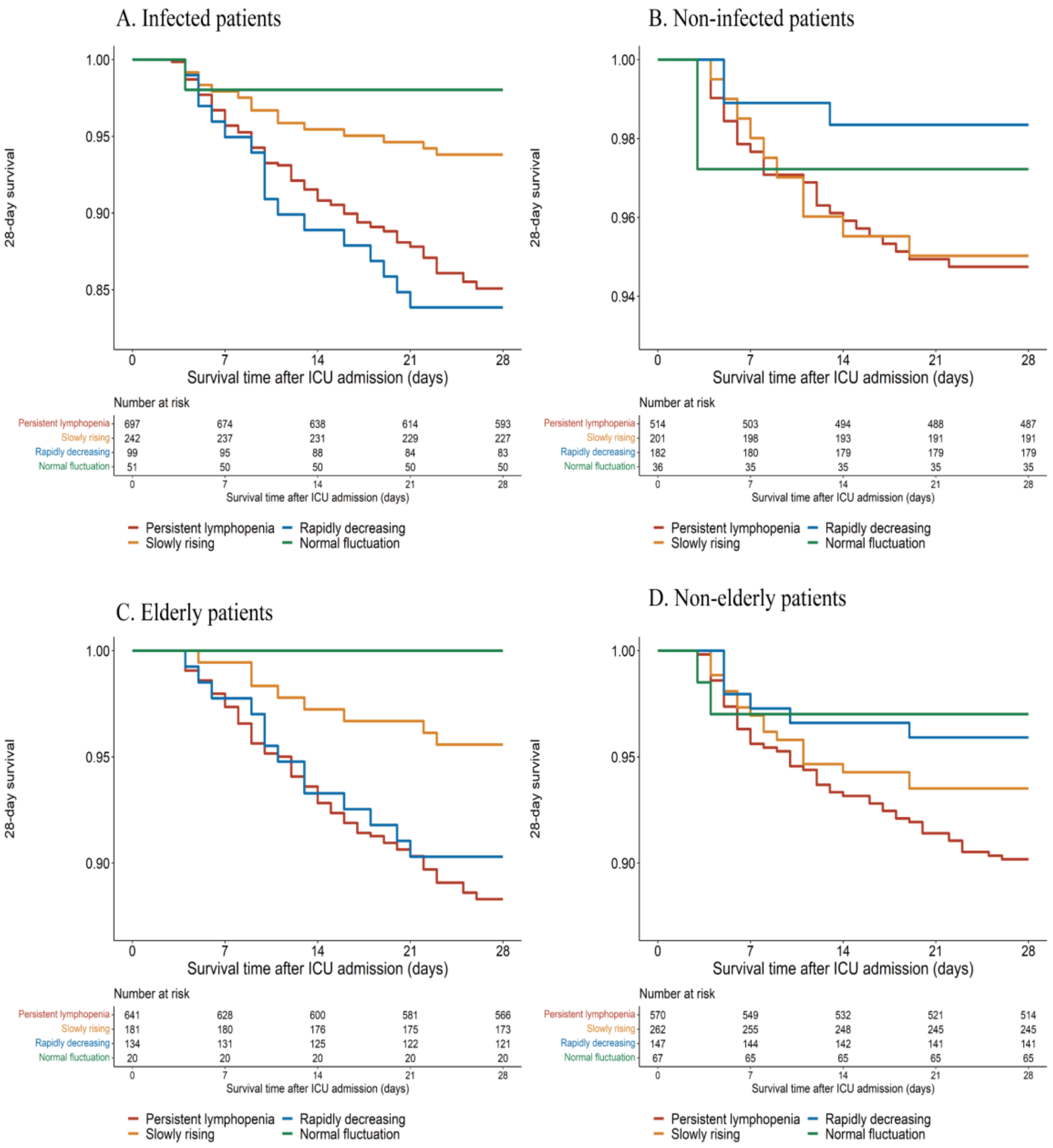


**Kaplan-Meier curves for 28-day mortality in different subgroups.** A. Infected patients; B. Non-infected patients; C. Elderly patients; D. Non-elderly patients.
